# RNA silencing can explain chlorotic infection patterns on plant leaves

**DOI:** 10.1186/1752-0509-2-105

**Published:** 2008-11-30

**Authors:** Marian AC Groenenboom, Paulien Hogeweg

**Affiliations:** 1 Theoretical Biology and Bioinformatics, University of Utrecht, Padualaan 8, 3584 CH Utrecht, the Netherlands

## Abstract

**Background:**

RNA silencing has been implicated in virus symptom development in plants. One common infection symptom in plants is the formation of chlorotic tissue in leaves. Chlorotic and healthy tissue co-occur on a single leaf and form patterns. It has been shown that virus levels in chlorotic tissue are high, while they are low in healthy tissue. Additionally, the presence of siRNAs is confined to the chlorotic spots and the boundaries between healthy and infected tissue. These results strongly indicate that the interaction between virus growth and RNA silencing plays a role in the formation of infection patterns on leaves. However, how RNA silencing leads to the intricate patterns is not known.

**Results:**

Here we elucidate the mechanisms leading to infection patterns and the conditions which lead to the various patterns observed. We present a modeling approach in which we combine intra- and inter-cellular dynamics of RNA silencing and viral growth. We observe that, due to the spread of viruses and the RNA silencing response, parts of the tissue become infected while other parts remain healthy. As is observed in experiments high virus levels coincide with high levels of siRNAs, and siRNAs are also present in the boundaries between infected and healthy tissue. We study how single- and double-stranded cleavage by Dicer and amplification by RNA-dependent RNA polymerase can affect the patterns formed.

**Conclusion:**

This work shows that RNA silencing and virus growth within a cell, and the local spread of virions and siRNAs between cells can explain the heterogeneous spread of virus in leaf tissue, and therewith the observed infection patterns in plants.

## Background

RNA silencing is an evolutionary conserved mechanism in eukaryotes that has a major role in gene regulation, development, transposon control and defense against viruses.

Antiviral RNA silencing is induced by virus double-stranded RNA (dsRNA) or by specific single-stranded RNA (ssRNA) structures. Double- or single-stranded RNA is cleaved into small interfering RNA (siRNA) by RNase III-like enzymes such as Dicer and Dicer-like [1,2]. siRNAs associate with the RNA-induced silencing complex (RISC) and guide the complex to complementary sequences that are then destroyed. In addition to the primary response, siRNAs can be produced through a secondary pathway that involves synthesis of dsRNA or siRNA by host encoded RNA-dependent RNA polymerase (RDR) [3-5].

The antiviral role of RNA silencing is extensively studied in plants [1,6,7]. Virus spread through the plant results in diverse symptoms, for example leaf curling, abnormal leaf or flower development, and patterns on infected leaves. These patterns consist of both chlorotic or necrotic tissue in combination with healthy looking tissue. Different types of patterns that occur are concentric circles or rings, mosaic patterns, vein clearing and spots. Interestingly, virus levels are high in yellow, chlorotic tissue and low in the green, healthy tissue [8]. This means that virus accumulation varies from cell to cell. It has been hypothesized that RNA silencing may play a role in the development of leaf patterns resulting from virus infections [9-12]. Recent observations by Hirai et al. [13] on mosaic patterns support this hypothesis. They have shown that RNA silencing activity is confined to the yellow spots and the marginal regions of the green spots. Reduced expression of RDR, which is part of the secondary pathway of the silencing response, resulted in smaller or no green tissue. These results strongly suggest that RNA silencing plays a major role in the development of plant symptoms.

Previously we developed a mathematical model of RNA silencing and its interaction with viral growth within a cell [14]. We found that depending on the strength of the silencing response the virus equilibrium can be almost unaffected, oscillations can occur, or the virus can be cleared. Additionally, we found that a change in Dicer cleavage rate is representative for a general change in silencing strength. For a low Dicer cleavage rate the equilibrium amount of virus is slightly decreased and the virus grows slower than without silencing. For high Dicer cleavage rate the virus is not able to grow and is cleared directly after introduction. For intermediate Dicer cleavage rates oscillations in virus levels can occur. When a secondary response is added these oscillations can be enlarged to such extend that the virus is cleared after a single growth peak.

We here study how RNA silencing can explain the development of leaf patterns resulting from viral infection. To this end we use a detailed modeling approach in which we combine an intra-cellular model of viral growth and the RNA silencing pathway with inter-cellular tissue dynamics.

We observe that RNA silencing and virus growth on a tissue can result in a discontinuous spread of the virus: the virus reaches high levels in some cells, while it is suppressed in other cells. We study the conditions for different type of patterns. These patterns could be the basis of plant symptom development. We elucidate the mechanisms leading to these patterns and how increased silencing efficiency, siRNA movement and the occurrence of a secondary response relate to the pattern formation.

## Methods

**Figure 1:**
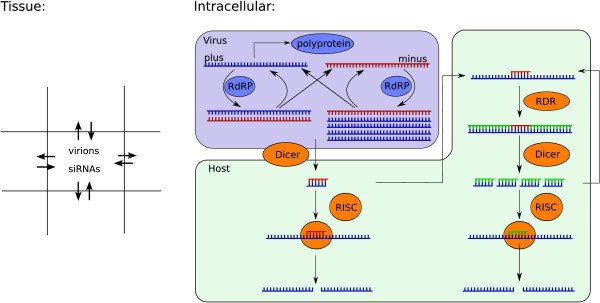
**Schematic representation of the model**. In each grid point the dynamics of a replicating plus-strand RNA virus and antiviral silencing are calculated. Viral plus- and minus-strand RNA is replicated by RdRP. The formed complex dissociates into ssRNA. Virus single-strand and part of the double-stranded RNA can be cleaved into siRNA by Dicer. siRNA associates with RISC and cleaves the target RNA. siRNAs can guide or prime amplification of the response through host encoded RDR, or viral ssRNA is amplified by RDR in an unprimed manner. The full intracellular model can be found in the Appendix. siRNAs and virions produced in each cell can move from cell to cell on the grid.

To investigate the formation of infection patterns in plants we model an area of plant tissue on a grid. Each grid point represents a plant cell. Within each cell we calculate virus levels and levels of RNA silencing proteins with a detailed model. Virions and siRNAs can move from cell-to-cell. A schematic representation of the model is shown in Figure 1.

### Intracellular model

The number of molecules in each cell is calculated with our previously described model of the antiviral RNA silencing pathway and a replicating plus-strand RNA virus [14].

The intracellular model consists of coupled differential equations, representing viral plus- and minus-strand RNA, dsRNA, virions, RdRP, siRNA targeting plus or minus-stranded RNA, and free and active RISC. The virus replication cycle starts with the translation of plus-stranded RNA into a poly-protein. After auto-cleavage one of the products is RNA-dependent RNA polymerase (RdRP) that associates with plus-strand RNA to synthesize a complementary strand. The formed dsRNA separates into a plus- and minus-strand that can both associate with RdRP again. We assume that the minus-strand is the preferred template for dsRNA synthesis. Semi-conservative synthesis of multiple plus-strands from a single minus-strand template is incorporated in the model, resulting in a biased plus-to-minus ratio. The virus produces virions that consist of plus-stranded RNA and coat proteins. We simplify here by using the number of plus-stranded RNA instead of modeling a separate coat protein.

Viral double- or single-stranded RNA is degraded by host encoded Dicer into siRNAs, that have a plus- or minus-strand polarity. siRNAs cleaved from dsRNA have a 50% chance of targeting either the plus or the minus strand. siRNAs cleaved from a ssRNA hairpin automatically target strands with the opposite polarity. Via RISC the siRNAs cause degradation of either plus- or minus-stranded viral RNA.

Secondary siRNAs can be synthesized through the amplification pathway that involves synthesis of dsRNA by host encoded RDR. We implement unprimed, primed and guided amplification. Each type of amplification can be studied separately.

All equations are integrated using a timestep of 3.6 seconds. Simulations run for 300 hours, unless indicated otherwise. The equations can be found in the Appendix.

### Tissue level model

To study RNA silencing and viral infection in a tissue we use a spatial model. Each grid point represents a cell for which the intracellular dynamics are calculated with the model described above.

Viruses encode movement proteins that enable the movement of virions from cell-to-cell through plasmodesmata. We implement the movement of virions to the four neighboring cells in our model. Virions can be unpacked in each cell into naked plus-strand RNA. We chose a 4 neighborhood because cells share almost no surface area with the diagonal neighbors.

For movement we shift to a particle based system, because we do not want incomplete particles to trigger a reaction in a neighboring cell. Movement of particles occurs every timestep (3.6 seconds). A fraction of the total number of virions in the cell is evenly distributed among the four neighbors, and excess virions are distributed randomly among the neighbors. When the number of moving virions is smaller than one, we draw a random number to decide if one virion moves to a random neighbor. With this method we underestimate the heterogeneity of viral spread as compared to Brownian motion. This method is therefore a good worst-case scenario for the study of heterogeneous spread of virus particles.

The silencing response is able to spread from cell-to-cell with a short range silencing signal, most likely siRNAs [15]. We implement the spread of siRNAs in the same way virions move. There is also a long-range silencing signal [16,17]. Because of the elusive nature of the long range silencing signal, and because we here take only a tissue or leaf area into account we do not include a long range silencing signal.

## Results and discussion

We vary Dicer cleavage rates as representative for silencing strength to study the effect of RNA silencing on the spread of virus particles over the tissue.

Within the cell three different behaviors can be observed [14]. High Dicer cleavage rate results in fast clearance of the virus. Low Dicer cleavage rate delays viral growth but hardly decreases the virus levels in equilibrium. Intermediate cleavage rate results in oscillating virus levels.

### Infection patterns without RNA silencing

We first study virus spread without silencing present. We initialize the tissue with healthy cells and infect one cell in the center with 10 viral plus strands. After initiation the virus starts to produce virions that spread from cell-to-cell. We fix the fraction of virions leaving a cell to 1% per hour.

**Figure 2:**
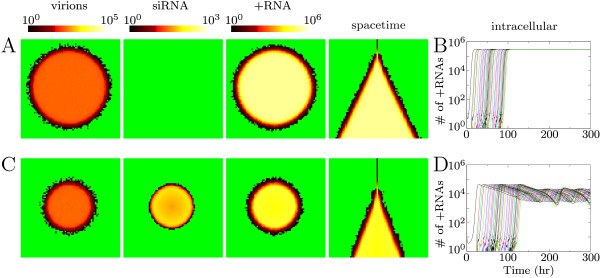
**Virus spread over plant tissue**. (A) and (C) are screen-shots 75 hours post infection showing the number of virions, siRNAs and +RNA in the cells. (B) and (D) show the intracellular +RNA levels for a row of cells through time. (A) and (B) show results without silencing and (C) and (D) with silencing. Virions move from cell-to-cell, but siRNAs cannot spread.

The virus spreads rapidly over the entire area and 75 hours post infection (hpi) a circular area of the grid has become infected with the virus. In Figure 2A we show the number of virions, the number of siRNAs and the number of plus strands in separate screen-shots. When virions, virus plus-stranded RNA or siRNAs are absent from the cell, it is shown in green. Cells that have virions, plus-stranded RNA or siRNAs are shown in a color ranging from black to yellow (via red): the color ramps are shown in Figure 2. Also shown is a space-time plot, that is, horizontal cross-sections of the grid every hour post infection (1 hpi top row, 75 hpi bottom row). In Figure 2B we show time-series of plus-strand accumulation in adjacent cells. The curve starting at 0 hpi is an initially infected cell. The other cells in turn each become infected by the neighboring cell(s) and in each cell the virus expands to the equilibrium.

### Infection patterns with silencing, without siRNA movement

When RNA silencing is active, we initialize the cells with RISC and Dicer. We first assume that Dicer is only capable of cleaving siRNA from viral dsRNA, and that siRNAs cannot move from cell to cell. Even without siRNA movement RNA silencing slows down virus spread over the area (Figure 2C). In the space-timeplot can be seen that the infection advances much slower than without the presence of the silencing response. A stronger silencing response slows down the spread of the virus more than a weaker response. We here show results for an intermediate Dicer cleavage rate, that results in oscillatory behavior within the cells (Figure 2D). For high Dicer cleavage rate the virus is cleared immediately after introduction, and is not able to spread from cell to cell, because it is eliminated before virions could be produced.

### Infection patterns with siRNA movement

As shown above, without siRNA movement the infection spreads homogeneously over the area. When siRNAs do move from cell to cell, they can limit virus growth in neighboring cells, resulting in viral growth in some cells and suppression in others. This results in patterns that can spread over the entire area or stay localized to the area around the inoculated site. We observe somewhat different patterns for low and intermediate Dicer cleavage rates and we will discuss results from both possibilities.

#### Low dsRNA cleavage rate by Dicer

We first study the effects of a low Dicer cleavage rate for all possible siRNA movement rates. Varying the rate of siRNA movement we observe that the virus does not spread uniformly over the area and that patterns are formed.

**Figure 3:**
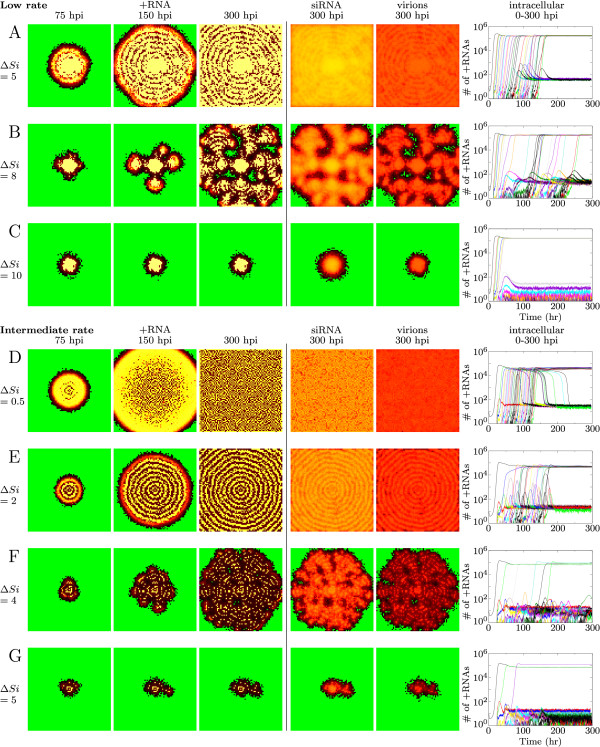
**Infection patterns caused by RNA silencing**. (A-C): low silencing strength (Dicer cleavage rate is 5 cleavages per Dicer per hour). (A) concentric circles, (B) mosaic and (C) local spot. (D-G): intermediate silencing strength (Dicer cleavage rate is 15 cleavages per Dicer per hour). (D) speckle-pattern; (E) concentric circles; (F) mosaic; and (G) local spot. The patterns spread over the entire tissue, except the patterns shown in C and G. In the first three columns the number of +RNAs is shown for 75, 150 and 300 hours post infection (hpi), column 4 and 5 show siRNA and virions, respectively. Also shown is the intracellular amount of +RNA through time for a row of cells. Δ*Si *gives the percentage of siRNAs that move to neighboring cells per hour. Colors according to the color-ramps shown in Figure 2.

Low siRNA movement results in a circular pattern that is shown in Figure 3A. The virus reaches a high equilibrium in the cells close to the initiation site. The siRNAs produced by these cells inhibit viral growth in a ring of cells around the center. Virions are unpacked in these cells, but the viral RNA is silenced with the siRNAs from the neighbors. At sufficient distance from the siRNA producing cells, the virus will be able to grow, and these cells will in their turn inhibit viral growth in the next ring. Once a ring is formed it remains stable.

In the time-series (Figure 3A) it can be seen that the virus can expand to an equilibrium with high RNA levels in some cells, while in other cells the virus decreases to low RNA levels. We will refer to these cells as "silenced cells". The silenced equilibrium is maintained by siRNA movement: the virus grows to the high equilibrium when siRNA movement is stopped. In silenced cells the siRNA influx suppresses virus replication completely: no dsRNA is formed, and the observed plus-strand RNA levels result from the virion influx from neighboring cells.

For a higher siRNA movement rate a different pattern occurs (Figure 3B). The circular pattern breaks and protrusive waves occur that resemble mosaic-like symptoms. We can distinguish three cell types in this pattern: cells with high virus levels, silenced cells and healthy cells. The healthy spots between the infected patches are surrounded by the silenced cells. Apart from low virus levels in the silenced cells at the edge of the spots there are no virions or other virus particles present in the healthy spots. The siRNAs present in the edge cells seem to "guard" the edges of the healthy spots as described by Hirai et al. [13]. These siRNAs, however, are not produced by the silenced cells in the edges, but move there from the infected neighboring cells.

For the maximum siRNA movement rate (10% of the available siRNAs in a cell) the infection is confined to the area around the inoculated site (Figure 3C).

Concluding, siRNA movement creates silenced cells in which the virus is suppressed, and cells in which the virus grows to high values. Increasing siRNA movement increases the number of silenced cells, rather than decreasing virus load in all cells.

#### Intermediate dsRNA cleavage rate by Dicer

When Dicer cleavage rate is intermediate, similar patterns can be observed (Figure 3D–G). However, a lower siRNA movement rate is sufficient to generate them. For low siRNA movement rate a speckle pattern occurs. Initially the virus is able to expand considerably in all cells (Figure 3D). However, some time after infection virus levels drop in some cells and reach the silenced equilibrium. After 300 hours the pattern is almost completely stable, either cells are at the high or at the low equilibrium. This speckle pattern distinguishes itself from the others by the high initial growth of the virus in all cells: in other patterns virus levels never reach this high levels before declining to the silenced state.

When siRNA movement increases concentric circles are formed as is the case for the lower silencing efficiency (Figure 3E). The number of silenced cells increases compared to the speckle pattern. A relatively low siRNA movement rate results in broad bands of virus infection, a higher rate results in very thin bands.

With still higher siRNA movement the thin bands in which the virus reaches high levels break, and a growing ice crystal-like pattern is observed (Figure 3F). The pattern consists of protrusive waves, and in only a small number of cells the virus reaches the high equilibrium. Because the infected spots are much smaller compared to the similar mosaic pattern for low silencing strength there are not enough siRNAs to create truly healthy spots.

With even higher siRNA movement rate the protrusive waves are reduced to a local spot, with the possibility of one or two small outbreaks (Figure 3G). For low silencing efficiency we also observed a spot pattern (Figure 3C). Low silencing efficiency results in a completely infected spot, while higher silencing efficiency results in a small ring-like pattern.

### Alternative equilibria depend on influx of siRNAs

As shown in the previous section, virus levels in the spatial model can reach two different equilibria, a high and a low one, while in the intracellular ODE model only a single equilibrium exists. The patterns disappear when siRNA movement is stopped, therefore siRNA movement maintains the silenced equilibrium. To analyze the influence of the tissue dynamics on the cellular dynamics mathematically we add in- and efflux of siRNAs and virions to the intracellular model. Efflux is fixed to the movement parameters used in the tissue model. We measure average virion and siRNA influx in the equilibrium for 4 cells from the center of the tissue and use these as influx values in the intracellular model.

**Figure 4:**
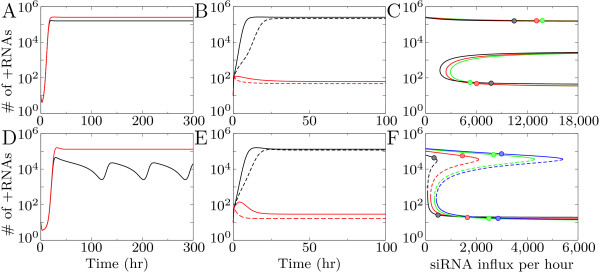
**The intracellular model with in- and efflux of siRNA and virions**. (A-C) low Dicer cleavage rate (5 cleavages per Dicer per hour); (D-F) intermediate Dicer cleavage rate (15 cleavages per Dicer per hour). (A) and (D) time-series for the intracellular model without in and efflux (black) and with efflux (red). (B) and (D) Time-series with in- and efflux showing bistability: low siRNA influx (black lines) results in a high equilibrium, high siRNA influx (red lines) in a low equilibrium. (C) and (F) Bifurcation diagram showing equilibrium values of viral plus-strand RNA for increasing siRNA influx. The different lines are calculated with a fixed virion influx measured for the different patterns in Figure 3. Δ*Si *in (C) from left to right lines: 5% (black), 8% (red), 10% (green); and in (F) from left to right: 0.5% (black), 2% (red), 4% (green), 5% (blue). Circles indicate the position of cells that reached the high and the low equilibrium in the tissue model.

As an example case we take the parameters and measurements for the mosaic-like pattern for low and intermediate silencing strength shown in Figure 3B and 3F. Without in- and efflux virus levels reach a high equilibrium for low silencing strength, and for intermediate silencing strength intracellular oscillations occur (black lines Figure 4A and 4D). Efflux of siRNAs and virions results in an increase in virus levels and the oscillations disappear (red lines in Figure 4A and 4D). When virion influx is fixed to the average virion influx measured and siRNA influx is added we observe a bifurcation: Depending on siRNA influx two equilibria occur, a high and a low one, corresponding to the equilibria in the spatial model (Figure 4B and 4E). A low siRNA influx results in virus growth to the high equilibrium, high siRNA influx results in growth to the silenced equilibrium.

We calculate bifurcation diagrams as a function of the siRNA influx. In Figure 4C we show the bifurcation diagram for low silencing strength. Each line in the bifurcation diagram is calculated with measurements from different patterns. For all measurements only the high equilibrium exists for low siRNA influx, while for higher siRNA influx there are three equilibria: two stable states separated by an unstable state (Figure 4C). Depending on the initial conditions and the siRNA influx the virus can either grow to the high or the low equilibrium. The number of virus RNAs in the high and low equilibrium are similar for the bifurcation lines calculated for different siRNA movement rates. However, the siRNA influx needed to reach the low equilibrium (that is, the bifurcation point) shifts to lower siRNA influx values for lower siRNA movement rate. Low siRNA movement rate increases the effect of silencing within the cell, because less siRNAs leave it and a lower siRNA influx is sufficient to reach the silenced state.

In the spatial model both virion and siRNA influx change over time. Therefore not the final siRNA influx but the combined virion and siRNA influx during the growth phase of the virus determine which equilibrium is reached. Once a stable state is reached a change in siRNA influx has no effect on equilibrium values. Only when siRNA influx is dramatically lowered or when a very large amount of virions would be introduced it would be possible to pass the bifurcation point and move from the silenced to the high state.

For intermediate silencing strength the bifurcation diagram is similar, however due to the lower siRNA efflux and the stronger silencing response within the cell, it is shifted toward lower siRNA influx. The high and the low equilibrium are connected, meaning that an increase in siRNA influx results in a shift from the high equilibrium to the low one. This is the case in the speckle pattern: all cells initially expand to the high equilibrium, but some cells decline to the silenced state later in infection.

To indicate the measured siRNA influx from the patterns shown in Figure 3, we placed circles in the bifurcation diagrams for two high and two low cells (Figure 4C and 4F). For most patterns the equilibrium values are far from the bifurcation points, resulting in a stable pattern unaffected by noise. For the speckle pattern the values are close to the bifurcation point. A slight increase in siRNA influx can push a cell with high virus levels to the silenced state. On the other hand a slight decrease in siRNA influx can result in growth from the silenced state to the high equilibrium. The resulting pattern is at a delicate balance: cells that reach the high equilibrium suppress virus growth in neighboring cells, and the silenced cells cause a low enough siRNA influx into the cell to stay at the high state.

Concluding, the effect of siRNA influx from neighboring cells is two-fold. Firstly, siRNA influx creates two coexisting equilibria; secondly, siRNA influx during the initial growth phase of the virus largely determines which equilibrium is reached.

### Combined single- and double-stranded RNA cleavage by Dicer

It has been proposed that Dicer can cleave ssRNA in addition to dsRNA [2]. On the cellular level combined single- and double-stranded cleavage by Dicer results in the diverse skewed siRNA ratios that have been observed [2,14,18,19]. Although combined Dicer cleavage of single- and double-stranded RNA can clear the virus for lower Dicer cleavage rates, it can also be less efficient. For the same total Dicer cleavage rate dsRNA cleavage results in somewhat lower plus-strand RNA levels then combined single- and double-strand cleavage [14]. This is due to the skewed siRNA ratio: many siRNAs are produced that target the minus-strand, but few siRNAs are produced that target the plus-strand. This bias results in slightly less efficient silencing, however when the minus strand is completely degraded, the virus is cleared despite the less efficient response.

The complex feedbacks between the intracellular and the spatial model result in an unexpected effect of ssRNA cleavage in the spatial model. While keeping total Dicer cleavage rate constant, increasing ssRNA cleavage by Dicer shifts the silenced equilibrium to higher RNA levels. An increase of virus levels in the silenced equilibrium results in less pronounced patterns and it can even result in disappearance of the silenced state.

**Figure 5:**
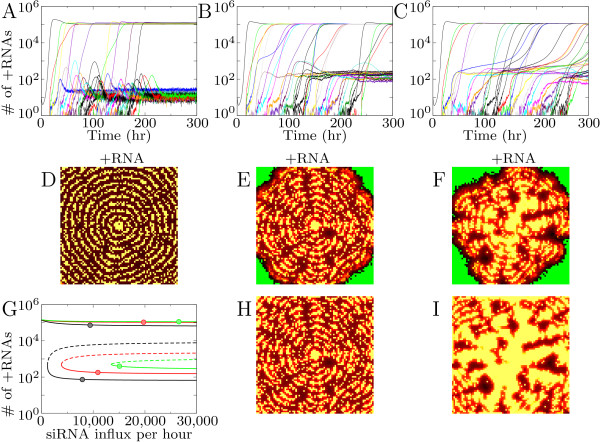
**Effect of ssRNA cleavage by Dicer on intracellular dynamics and infection patterns**. Total Dicer cleavage rate is 10 cleavages per Dicer per hour and Δ*Si *= 4%. (A-C) measured intracellular +RNA levels from spatial model with Dicer cleavage rates: (A) 10 on dsRNA, 0 on ssRNA; (B) 8.5 on dsRNA, 1.5 on ssRNA; (C) 8.05 on dsRNA, 1.95 on ssRNA. (D-E) The infection patterns corresponding to (A-C). Shown are +RNA levels 300 hours post infection. (G) Bifurcation diagram of intracellular +RNA levels for increasing siRNA influx. The different lines are calculated with parameters from (A) black; (B) red; and (C) green. Circles indicate the position of 2 cells from the spatial model that reached the high or low equilibrium. (H and I) infection patterns showing +RNA levels corresponding to (B) and (C) 450 hours post infection. Colors according to the color-ramps in Figure 2.

In Figure 5D–F we show patterns for increasing rates of ssRNA cleavage by Dicer 300 hpi. The patterns change from circles to mosaic-like, and the silenced cells change from a dark to a red color, indicating higher virus levels in silenced cells. This effect becomes more clear later in infection (Figure 5H). Moreover, the infection pattern shown in Figure 5F fades out (at 450 hpi, shown in Figure 5I), and eventually the entire tissue becomes fully infected.

In Figure 5A–C we show how the equilibria change when changing ratio's of double-to single-stranded RNA cleavage by Dicer. We keep total Dicer cleavage rate constant at 10 cleavages per Dicer per hour. To explain the observed siRNA ratios approximately 0–20% of Dicer cleavages should take place on ssRNA [14]. In Figure 5A 5% of Dicer cleavages is on ssRNA and in Figure 5B 15%. The silenced state clearly shifts to higher virus levels when ssRNA is cleaved. When more ssRNA is cleaved the equilibrium shifts further upwards until it disappears. In Figure 5C we show the behavior for 19.5% ssRNA cleavage by Dicer, at 20% ssRNA cleavage the silenced state has completely disappeared. The upwards shift of viral RNA levels implies that virus replication is not completely silenced. Indeed dsRNA is produced in the silenced cells.

These results are explained further in the bifurcation diagram in Figure 5G. Shown are the equilibrium values of plus-strand RNA as a function of siRNA influx. The different lines represent a varying fraction of ssRNA cleavage by Dicer. The virion influx increases for increasing ssRNA cleavage by Dicer and the silenced state shifts upwards. Additionally, an increasingly higher siRNA influx is needed to reach this equilibrium. The dots indicate the location of cells from the spatial model that reach the high and the low equilibrium. Due to increased siRNA levels the siRNA influx is increased when ssRNA is cleaved by Dicer. However, due to the less efficient silencing within cells, an increasingly higher siRNA influx is needed to reach the silenced state.

This means that, although combined single- and double-stranded RNA cleavage by Dicer can clear the virus at lower Dicer cleavage rate [14], the slight increase in virus equilibrium is disadvantageous at the tissue level as RNA silencing can no longer fully suppress virus replication in silenced cells. However, an advantage of single strand cleavage by Dicer is the slower rate of spread over the tissue.

### Effect of amplification

Amplification of silencing through RDR can decrease viral levels in plants [20-22]. It also affects symptoms observed: plants without a functional RDR become fully infected or die, while plants with RDR show mild chlorosis or mosaic [23,24].

**Figure 6:**
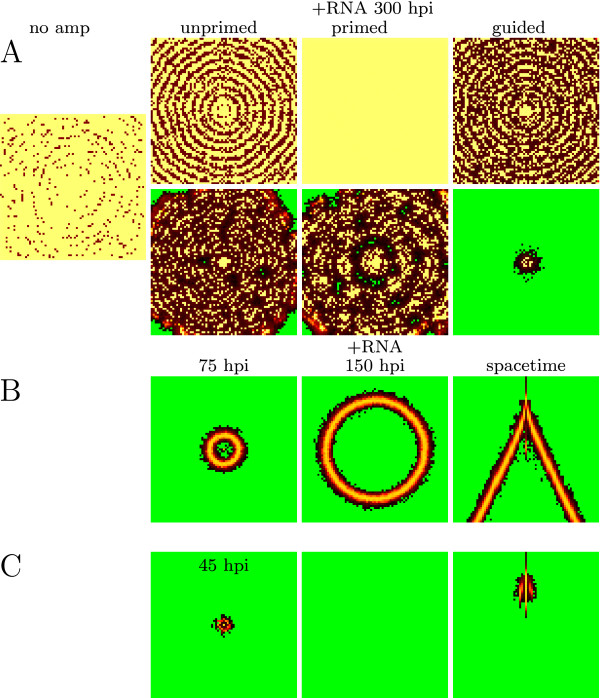
**Infection patterns with different types of amplification**. (A) low double-strand Dicer cleavage rate (5 cleavages per Dicer per hour). Top row shows screen-shots for 1 siRNA per amplified transcript, bottom row 4 siRNAs per amplified transcript. siRNA movement is 4%. Amplification can reduce virus infection to mosaic or circle patterns, while it infected almost the entire tissue without amplification. (B-C) New patterns for intermediate double-strand Dicer cleavage rate (15 cleavages per Dicer per hour) and amplification. (B) zero siRNA movement results in a growing circle that leaves uninfected tissue behind; (C) 5% siRNA movement results in the appearance and disappearance of a spot. Parameters used: (B) primed amplification with 4 siRNAs per amplified transcript; (C) guided amplification with 4 siRNAs per amplified transcript. Colors according to the color-ramps shown in Figure 2.

To study the effect of the secondary response on pattern formation and virus levels we add amplification to the model. At the cellular level RNA silencing with unprimed amplification can clear the virus for much lower silencing strength. It has to be noted however, that unprimed amplification can be triggered by any RNA, and will lead to responses against host RNA. Therefore, a mechanism has to be included to protect the host against auto-immunity [25,26]. Primed and guided amplification can increase oscillations and create a new region of behavior, in which the virus is degraded after an initial growth peak. Primed amplification can only be beneficial when there is a net siRNA gain [14]. At low Dicer cleavage rate the virus equilibrium is slightly decreased on the cellular level. In the spatial model the addition of amplification can result in a change of patterns (Figure 6A). Without amplification almost the complete area becomes infected with the virus, with amplification circles or mosaic-like patterns can occur.

Concluding, for low silencing strength amplification has a similar effect to increasing silencing strength or siRNA movement.

New patterns can be observed when the virus is cleared after an initial growth peak at the cellular level. Because the virus can expand and produce virions initially, it is able to spread over the tissue. However, in each cell the virus will be completely degraded by the RNA silencing response. We therefore observe transient patterns that leave healthy tissue behind. These patterns are shown in Figure 6B and 6C. Without siRNA movement a single growing ring occurs, low siRNA movement can lead to a disappearing spot near the infected site. These results indicate that a strong feedback can result in transient infection patterns. When siRNA movement is higher, the feedback becomes smaller due to the efflux of siRNAs, and infection patterns as shown in Figure 3 and 6A occur.

## Conclusion

We have shown that RNA silencing causes local differences in virus accumulation that can be the basis of different virus symptoms developed in plants. We have studied all qualitatively different patterns that can occur for different parameter values.

When siRNAs spread from cell to cell, we observe patterns that can spread over the entire tissue and consist of alternating healthy and infected tissue. When siRNAs are able to spread quickly we observe localized spots around the infection site. The presence of a secondary response can result in transient patterns that leave healthy tissue behind. In accordance with our results, it has been shown that in absence of a secondary response tissue can get fully infected, while with secondary response patterns develop [23,24]. The initial appearance of patterns that slowly fade until the entire tissue is infected occurs only when ssRNA is cleaved by Dicer.

In plants a variety of chlorotic patterns caused by viral infection have been observed. Chlorotic tissue contains high virus levels [27]. Some of the patterns of our model resemble infection patterns in plants. When silencing is weak the entire leaf area becomes infected, corresponding to chlorosis of the entire leaf.

When silencing is stronger we observe a mosaic-like pattern that resembles symptoms observed for many mosaic viruses. A difference with the experimentally observed patterns and our simulated patterns is the scale. We only observe larger scale mosaic when silencing strength is low. When silencing strength is high or when the secondary response is active we observe a small scale mosaic pattern. Our model, however, does not include growth of the tissue, and it has been shown that the healthy spots in mosaic-like patterns come about from cell division of single cells or a cluster of a few cells (reviewed in [27]). Development and growth of our small scale mosaic-like pattern could result in the experimentally observed large scale patterns.

In our model local spots occur when siRNA movement and silencing strength are sufficiently high. The virus infection stays localized to the area around the initially infected cell. We observe two types of spots: spots that are completely infected and ring spots that have a circle of low virus levels in between high levels. Lesions and ring spots are very common local symptoms after inoculation.

Ring patterns similar to our concentric rings have been observed for Tomato ringspot virus [28]. Ringspot viruses can also give rise to a large single ring-like spot that resembles the single growing ring that we observed. Tissue at the leading edge of virus infection is infected, tissue in the center of the ring is healthy. We observe this pattern only when there is a very strong feedback, as for example a secondary response. In contrast to our rings, the observed ring patterns do not spread over the entire tissue. This could be due to a long range silencing signal that we have not included in our model. The effect of such signal could be tested by adding an influx of siRNA or dsRNA at a specific site on the grid. This site represents the presence of the vascular system of the plant through which the silencing signal spreads. In this way the effect of different candidates for the long-range silencing signal could be tested.

We have used a detailed model of both intra- and inter-cellular dynamics of virus replication and RNA silencing. Nevertheless we were able to analyze mathematically (by bifurcation diagram) the intracellular dynamics that lead to alternative equilibria underlying the formation of infection patterns in plants. We have shown that siRNA movement is the driving force behind the pattern formation observed. Data of Hirai et al. [13] on siRNA location strongly support our model. Further experiments and parameter validation is necessary to study specific cases, and we hope to inspire researchers to further investigate how the chlorotic patterns on plant leaves relate to virus and siRNA levels. Additionally, it would be very interesting to study the development of these patterns and local virus and siRNA levels in time-series.

In conclusion, we have shown that the interplay of RNA silencing and virus growth within a cell, and the spread of virions and siRNAs between cells can explain the variety of viral infection patterns observed in plants.

## Authors' contributions

MG and PH conceived and designed the models, and wrote the paper. MG performed the numerical computations. All authors read and approved the final manuscript.

## Appendix

Intracellular dynamics

The entire intracellular model:

(1) $$\begin{array}{ll} \text{RdRP} & \frac{dR}{dt}=\frac{rP}{\left(P+k_{t}\right)}-d_{r}R-\{o(1-f)P+ofM+o_{d}D_{m}\}F+hD_{p}+hR_{a}+\\ & G_{d}(D_{p}+R_{a}) \end{array}$$

(2) $$\begin{array}{ll} +\text{RNA} & \frac{dP}{dt}=-o(1-f)PF+hD_{p}+hR_{a}-dP-\frac{vP^{5}}{k_{v}^{5}+P^{5}}-\frac{b_{2}R_{m}P}{P+k_{ri}}-G_{p,m}P\\ & -\frac{b_{2}R_{sm}P}{P+k_{ri}}-A_{u}P-A_{p}(Si_{m}+Sis_{m})P-A_{g}(Si_{m}+Sis_{m})P \end{array}$$

(3) $$\begin{array}{ll} -\text{RNA} & \frac{dM}{dt}=-ofMF+hD_{p}+hD_{m}{\left(1-\frac{1}{D_{m}}\right)}^{\left(R_{a}-D_{m}\right)}-dM-\frac{b_{2}R_{p}M}{M+k_{ri}}\\ & -G_{p,m}M-\frac{b_{2}R_{sp}M}{M+k_{ri}}-A_{u}M-A_{p}(Si_{p}+Sis_{p})M-A_{g}(Si_{p}+Sis_{p})M \end{array}$$

(4) $$\begin{array}{cc} \text{Virions} & \frac{dV}{dt}=\frac{vP^{5}}{k_{v}^{5}+P^{5}}-d_{v}V \end{array}$$

(5) $$\begin{array}{cc} \text{dsRNA} & \frac{dD_{p}}{dt}=o(1-f)PF-hD_{p}-G_{d}D_{p} \end{array}$$

(6) $$\begin{array}{cc} \text{dsRNA} & \frac{dD_{m}}{dt}=ofMF-hD_{m}{\left(1-\frac{1}{D_{m}}\right)}^{\left(R_{a}-D_{m}\right)}-G_{d}D_{m} \end{array}$$

(7) $$\begin{array}{cc} \text{act}\text{.RdRP} & \frac{dR_{a}}{dt}=ofMF+o_{d}D_{m}F-hR_{a}-G_{d}R_{a} \end{array}$$

(8) $$\begin{array}{cc} +\text{siRNA} & \frac{dSi_{p}}{dt}=G_{p,m}P+0.5\;G_{d}(D_{p}+D_{m})-d_{si}Si_{p}-b_{1}Si_{p}R_{f}-A_{p}Si_{p}M \end{array}$$

(9) $$\begin{array}{cc} -\text{siRNA} & \frac{dSi_{m}}{dt}=G_{p,m}M+0.5\;G_{d}(D_{p}+D_{m})-d_{si}Si_{m}-b_{1}Si_{m}R_{f}-A_{p}Si_{m}P \end{array}$$

(10) $$\begin{array}{cc} \text{free RISC} & \frac{dR_{f}}{dt}=i-d_{r}R_{f}-b_{1}R_{f}(Si_{p}+Si_{m}) \end{array}$$

(11) $$\begin{array}{cc} +\text{RISC} & \frac{dR_{p}}{dt}=b_{1}R_{f}Si_{p}-d_{r}R_{p} \end{array}$$

(12) $$\begin{array}{cc} -\text{RISC} & \frac{dR_{m}}{dt}=b_{1}R_{f}Si_{m}-d_{r}R_{m} \end{array}$$

(13) $$\begin{array}{cc} \text{dsRNA amp} & \frac{dD_{e}}{dt}=A_{u}(P+M)+A_{p}((Si_{m}+Sis_{m})P+(Si_{p}+Sis_{p})M)+\\ & A_{g}((Si_{m}+Sis_{m})P+(Si_{p}+Sis_{p})M)-G_{d}D_{e} \end{array}$$

(14) $$\begin{array}{cc} \sec.+\text{siRNA} & \frac{dSis_{p}}{dt}=0.5\;G_{d}D_{e}-d_{si}Sis_{p}-b_{1}Sis_{p}R_{f}-A_{p}Sis_{p}M \end{array}$$

(15) $$\begin{array}{cc} \sec.-\text{siRNA} & \frac{dSis_{m}}{dt}=0.5\;G_{d}D_{e}-d_{si}Sis_{m}-b_{1}Sis_{m}R_{f}-A_{p}Sis_{m}P \end{array}$$

(16) $$\begin{array}{cc} \sec.+\text{Risc} & \frac{dR_{sp}}{dt}=b_{1}Sis_{p}R_{f}-d_{r}R_{sp} \end{array}$$

(17) $$\begin{array}{cc} \sec.-\text{Risc} & \frac{dR_{sm}}{dt}=b_{1}Sis_{m}R_{f}-d_{r}R_{sm} \end{array}$$

The biological meaning of the variables is mentioned to the left of the equations. Multiple RdRPs can bind to minus-strand RNA, we refer to these as 'active RdRPs'. +RISC and -RISC are RISC loaded with siRNA with a plus- or minus-strand polarity. The abbreviation 'sec.' stands for secondary and is used to indicate siRNA that is produced through a secondary amplification pathway. Secondary RISC is loaded with secondary siRNA.

All parameter values can be found in Table 1, as well as a short description of each parameter. The other terms F, G and A are functions for the complex formation between RdRP and RNA strands, Dicer cleavage and amplification, respectively.

**Table 1:** Parameter values used. #mol is number of molecules.

**Model**	**Par**.	**Meaning**	**Value**	**units**
Intracellular	*r*	maximum translation rate × #ribosomes	15*5000	#mol hr^-1^
	*o*	max rate of complex formation ssRNA	1	hr^-1^
	*o*_*d*_	max rate of complex formation dsRNA	100	hr^-1^
	*f*	ratio of binding plus or minus RNA	0.9	-
	*h*	dsRNA-RDR splitting rate	10	hr^-1^
	*v*	max virion production rate	500	#mol hr^-1^
	*D*_*i*_	number of Dicer molecules	500	#mol
	*c*_*d*_	max Dicer cleavage rate for dsRNA	0–15	#mol hr^-1^
	*c*_*s*_	max Dicer cleavage rate for ssRNA	0–10	#mol hr^-1^
	*b*_1_	rate of RISC activation	0.005	#mol ^-1 ^hr^-1^
	*b*_2_	RISC target cleavage rate	20	#mol ^-1 ^hr^-1^
	*i*	translation of RISC	100	#mol hr^-1^
	*a*	amplification (*a*_*u*_, *a*_*p *_and *a*_*g*_)	100	#mol hr^-1^
	*d*_*r*_	decay RdRP and RISC	0.1	hr^-1^
	*D*	decay viral ssRNA	0.5	hr^-1^
	*d*_*si*_	decay siRNA	2	hr^-1^
	*d*_*v*_	decay virions	0.1	hr^-1^
	*k*_*v*_	saturation of virion production	10,000	#mol
	*k*_*d*_	saturation of Dicer cleavage	10,000	#mol
	*k*_*t*_	saturation constant for translation	1,000	#mol
	*k*_*ri*_	saturation of RISC cleavage	1,000	#mol
	*k*_*r*_	saturation of complex formation	1,000	#mol
	*k*_*a*_	saturation amplification	1000	#mol
Spatial	Δ*Si*	percentage of siRNAs exiting the cell	0–10	hr^-1^
	Δ*V*	percentage of virions exiting the cell	1	hr^-1^
	*u*	unpacking rate of virions	2	#mol hr^-1^

The complex formation (F) between RdRP and RNA strands is saturated for both viral RNA and RdRP (the Beddington-DeAngelis functional response [29,30]):

(18) $$F=\frac{oR}{R+P+M+D_{m}+k_{r}}$$

Dicer can cleave double-stranded and single stranded RNA and is saturated for *D*_*e*_, *D*_*p*_, *D*_*m*_, *P *and *M*. The Dicer cleavage functions, one for cleaving dsRNA and one for ssRNA, are saturated according to the ratio between single- and double-stranded RNA cleavage (*q*) by Dicer:

(19) $$\begin{array}{c} G_{d}=\frac{(1-q)c_{d}D_{i}}{(1-q)(D_{p}+D_{m}+D_{e})+q(P+M)+k_{d}} \end{array}$$

(20) $$G_{s}=\frac{qc_{s}D_{i}}{(1-q)(D_{p}+D_{m}+D_{e})+q(P+M)+k_{d}}$$

The amplification terms are:

(21) $$A_{u}=\frac{a_{u}}{\left(P+M+k_{a}\right)}$$

(22) $$A_{g}=A_{p}\frac{a_{p,g}}{\left((Si_{p}+Sis_{p})M+(Si_{m}+Sis_{m})P+k_{a}\right)}$$

Where $A_{u}$ is unprimed amplification, $A_{p}$ is primed amplification and $A_{g}$ is guided amplification. We study the amplification pathways separately. In the case of guided amplification, the siRNAs are not removed when they guide amplification, in contrast to primed amplification. Amplification produces dsRNA that is not used for virus replication (*D*_*e*_). This dsRNA is degraded into secondary siRNAs with a plus- or minus-strand polarity; *Sis*_*p *_and *Sis*_*m *_respectively.

### Parameters

For the intracellular dynamics we use the parameters previously described [14]. Parameter values can be found in Table 1. The only intracellular parameter that we vary in this study is the cleavage rate by Dicer (0 to 15 cleavages per Dicer per hour), because it is representative for a general increase in strength of the silencing response [14].

The extra parameters of the spatial model are the percentages of siRNAs and virions that move out of a cell (0 to 10% per hour), and virion unpacking rate.
